# A Medical Causerie

**Published:** 1915-05-15

**Authors:** 


### A MEDICAL CAUSERIE.

Even yet, it would appear, there is some uncertainty in reference to the exact requirements of the War Office relative to the medical profession. More than one deputation has recently visited Whitehall, and the proposals are not yet exhausted. Where the fault lies, if fault there be, it is not easy to say; but what is certain is that Sir Alfred Keogh placed beyond doubt the immediate necessity for large numbers of the younger men prepared to take commissions and serve with the troops in the field. This is the urgent claim, and upon this point misunderstanding is not possible. How far it is being met there is at present no means of knowing, but the reputed leaders of the profession do not show much evidence of activity in the matter. Other arrangements are necessary, but are by no means so acute. Thus the staffing of the new military hospitals which are to be established in London is likely to make considerable demands on the neighbouring general practitioners, but the organisation of the scheme is yet in an early stage. So far it has not been considered necessary to appoint consulting physicians and surgeons to these hospitals; and as this will avoid further interference with the work of the civil hospitals, it has obvious advantages. But what exactly will be needed will only be discovered when the hospitals get to work. The point remains—Are doctors being found for the new armies?

The decision to cancel the annual meeting of the British Medical Association was announced some weeks ago. The meeting was to have been held at Cambridge, with Sir Clifford Allbutt as President; and it is now proposed that all the contemplated arrangements shall hold good for 1916. This exhibition of faith in the favourable fortunes of the war is to be welcomed, and it is to be hoped that the event will justify it. The old lady who, in a period of extreme drought, attended a prayer meeting for rain armed with a substantial umbrella, set an example which is much to be commended. The annual report of the Association, in which the above intimation is made, is a truly formidable document and a chronicle of many activities. If members have time to read it through they must be much less occupied than rumour asserts. A rather unhappy feature of the report is an announcement that the membership has declined by over two thousand, and this, following a similar decline in 1913, brings down the total to 22,201. A somewhat naïve remark is the announcement that the Council has received many favourable comments on the reduction in the bulk of the Journal.

The death of Sir William Gowers removes a well-known figure from the profession in London. The abiding value of his work has been widely recognised, and success never quenched his zeal. Associated very prominently with diseases of the nervous system he still kept a catholic outlook on medicine generally, and he was very far indeed from the point of view of the "mere neurologist." Shorthand and medical ophthalmology were two of his pet subjects, and some years ago he founded the Society of Medical Phonographers, which, for a time, published a medical journal printed in appropriate character. In the general habit of his mind there was a strong element of independence, and he did not trouble to accommodate himself to methods which often lead to popularity. His teaching was not always easy to follow, but to those who took it seriously there were substantial rewards, and many grateful pupils will treasure his memory with admiration and respect.

There is a special interest in the award by the Ophthalmological Society of the Nettleship medal and prize to Mr. Treacher Collins. Apart from the valuable work done by Mr. Collins in the department of ophthalmology the recognition is peculiarly appropriate, as it is very largely due to him that the society has continued to exist. Two or three years ago there was an energetic agitation in favour of dissolution for the purpose of forming an ophthalmological section in the Royal Society of Medicine. After a decided opposition, more especially from the country members, the proposal was on the point of being carried, when Mr. Collins pointed out that it was quite possible to form a new Ophthalmological Section without destroying the Ophthalmological Society. The former was mainly a London interest, but the latter concerned the whole of the United Kingdom. Acting on this suggestion the society now leaves the frequent meetings at which cases, specimens, etc., are exhibited to the Royal Society, and continues its own existence in the shape of an annual congress at which an organised programme extending over three consecutive days is submitted. The plan has been very successful, and it was a happy arrangement which allowed a special compliment to Mr. Collins at the recent London meeting.

The new Red Cross hospital near Waterloo Station is now nearly ready for occupation. It is intended to accommodate 1,650 patients, and the equipment of it must have been a most expensive undertaking. Indeed, there is a suggestion that the society has hardened its heart against any further outlay, for an appeal is issued to generous-hearted persons for "the loan of five microscopes," which the authorities "will do their best" to return when the hospital is closed. The instruments are to be duly equipped with "a half- and a quarter-inch objective." Evidently they mean to get to the heart of things in this new institution. All the same, the image of the ship which was spoiled for want of "a hap'orth of tar" crosses the mind.

As the outcome of a recent discussion noted in The Hospital of April 10, a committee has been engaged in compiling a list of the balneological stations of the United Kingdom and of the treatment they can furnish for wounded and injured soldiers. This list will be submitted to the medical authorities of the Army, and it may be hoped that the facilities offered will be widely utilised.

